# High‐Speed Slot‐Die Coating with Donor‐Priority Rapid Aggregation Kinetics for Improved Morphology and Efficiency in Ecofriendly Organic Solar Cells

**DOI:** 10.1002/advs.202502077

**Published:** 2025-04-26

**Authors:** Zhaozhao Bi, Baohua Wu, Ke Wang, Jingwei Xue, Chang Liu, Lingxiao Tang, Ke Zhou, Long Jiang, Wei Ma

**Affiliations:** ^1^ State Key Laboratory for Mechanical Behavior of Materials Xi'an Jiaotong University Xi'an 710049 China; ^2^ Tubular Goods Research Institute of CNPC Xi'an 710049 China

**Keywords:** high‐speed printing, molecular aggregation kinetics, morphology engineering, non‐halogenated solvent, slot‐die coating

## Abstract

Solution‐processable organic solar cells (OSCs) represent a promising renewable photovoltaic technology with significant potential for eco‐compatible production. While high power conversion efficiencies (PCEs) have been achieved in OSCs, scaling this technology for high‐throughput manufacturing remains challenging. Key reason lies in the lack of efficient control strategies for the complex and long‐duration morphology evolution during high‐speed coating process with ecofriendly solvents. Here, a donor‐priority rapid aggregation process (DP‐RAP) scheme is proposed to solve this issue by adjusting the aggregation kinetics of donor and acceptor components. DP‐RAP enables blends with a nanoscale fiber network structure and favorable crystallinity, which contributes to balanced carrier transport and reduced recombination losses. As a result, the PCE is improved from 14.3% (reference) to 17.4% (DP‐RAP) for ultra‐high speed coated PM6:BTP‐eC9 devices in atmosphere, which is one of the highest values for non‐halogenated solvent‐processed solar cells at coating speeds of 500 mm s^−1^. Moreover, the DP‐RAP based devices remain a stable PCE of approximately 17.4% across a broad range of coating speeds (20–500 mm s^−1^), illustrating its tolerance to the varied manufacturing conditions. This work highlights a promising avenue for the high‐speed, ecofriendly production of efficient OSCs, pushing the boundaries of practical manufacturing in renewable energy technologies.

## Graphical abstract

A donor‐priority rapid aggregation strategy is developed to efficiently improve the film‐forming kinetics as well as film morphology. The resultant slot‐die coated ecofriendly organic solar cells exhibit high power conversion efficiency of 17.4% at ultrahigh coating speed of 500 mm s^−1^, without the need for additives or complex post‐treatment.

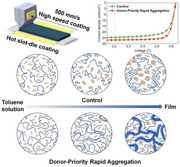

## 1 Introduction

Solution‐processable organic solar cells (OSCs) are emerging as a promising energy source for applications such as building‐integrated photovoltaics and self‐powered flexible devices, owing to their lightweight, flexibility, and semi‐transparency.^[^ ^1^, ^2^ ^]^ Currently, with the development of fused‐ring electron acceptor (FREA), especially the Y6 series and derivatives, the power conversion efficiency (PCE) of spin‐coated OSCs has surpassed 20%, paving the way for commercialization.^[^ ^3^, ^4^, ^5^ ^]^ Despite the prominent progress in device efficiency, three critical issues still need to be addressed for the scalable manufacturing of OSCs. First, the most common used techniques for producing high‐performance active layers are of low process capability (e.g., spin coating or conventional low‐speed blade coating), which is not comply with the required technical requirements for convenient scaling into high‐throughput, fab‐scale manufacturing.^[^ ^6^, ^7^, ^8^ ^]^ Second, the state‐of‐the‐art OSCs are typically processed with toxic halogenated solvents with low‐boiling‐point (like chloroform), which usually needs a strict inert atmosphere and poses significant health and environment risks.^[^ ^9^, ^10^ ^]^ Third, due to the complex donor (D) and acceptor (A) aggregation kinetics during the formation of bulk heterojunction (BHJ) films, the aggregation kinetics and final morphology are highly dependent on the fabrication conditions, which may result in unfavorable performance fluctuations and poses limitations to continuous production.^[^ ^11^, ^12^ ^]^

Spin coating is an effective method for the deposition of photoactive layers in laboratory scale, but it does not meet the requirements of fab‐scale, continuous and high‐throughput fabrication. Meniscus‐guided coating, such as blade coating and slot‐die coating, is compatible with large‐scale and high‐speed manufacturing and therefore are expected to be used in the commercial production of OSCs.^[^ ^13^, ^14^, ^15^, ^16^ ^]^ In particularly, the slot‐die coating enables precise control of solution and substrate temperatures, facilitating effective kinetics regulation in the atmosphere with no dependence on complex post‐treatments,^[^ ^17^, ^18^, ^19^ ^]^ which is important for the simplification of high‐throughput production process. Despite the significant benefits of high linear speed printing in terms of high throughput and low production costs, however, high‐speed solution printing that is compatible with high‐throughput roll‐to‐roll (R2R) coating technology has been rarely reported.^[^ ^7^, ^20^ ^]^ It is well known that the increase in coating speed will lead to an exponential increase in wet film thickness, which is known as the Landau‐Levich regime.^[^ ^21^, ^22^, ^23^ ^]^ In this case, the film drying process will be significantly prolonged, which can result in excessive molecular aggregation and large‐scale phase separation, and is detrimental to morphology optimization.^[^ ^23^, ^24^ ^]^ Thus, it is crucial to develop facile strategy to control morphology formation kinetics under high‐speed coating conditions.

To approach industrial production, ecofriendly solvents are imperative for the replacement of the lab‐used toxic solvents. Hydrocarbon solvents, known for being less toxic than the halogenated counterparts, are gradually being utilized to dissolve π‐conjugated photoactive materials.^[^ ^25^, ^26^ ^]^ However, the limited solubility of donor and acceptor materials in these solvents often results in significantly different active layer morphologies, posing challenges for performance enhancement of eco‐friendly OSCs.^[^ ^27^ ^]^ Recently, progress has been made in the non‐halogenated solvents processed OSCs thanks to the improvements in molecular design (e.g., alkyl chain engineering) and coating techniques (e.g., hot blade coating and hot slot‐die coating).^[^ ^28^, ^29^, ^30^ ^]^ These achievements have significantly promoted the practical manufacturing of eco‐friendly OSCs forward. Of note is that most of the improvements were achieved by spin coating or low‐speed printing (below 50 mm s^−1^), and research on eco‐friendly devices fabrication under high‐speed printing is still relatively rare. Meanwhile, the high boiling point and slow evaporation features of non‐halogenated solvents will inevitably prolong the film drying time. This will further prolong the film formation time for non‐halogenated solvents‐based high‐speed coating and make it difficult to process blend films with rational morphology.

For the conventional BHJ‐based devices, the film drying process involves the aggregation and crystallization kinetics of donor and acceptor as well as the phase separation process between donor and acceptor, which finally contributes to a complex interpenetrating donor‐acceptor framework for efficient exciton dissociation and balanced carrier transport.^[^ ^31^, ^32^, ^33^ ^]^ Therefore, many meticulous strategies have been developed to control the film formation kinetics (e.g., temperature‐mediated aggregation control, airflow‐assisted evaporation regulation, composition‐dependent phase separation control, etc.).^[^ ^34^, ^35^, ^36^, ^37^ ^]^ These researches have greatly promoted the process of morphology optimization and performance improvement. However, the dependence of donor and acceptor aggregation process on the manufacturing factors has rarely been concerned. Particularly, the different solubility of non‐halogenated solvents for donors and acceptors would further induce different aggregation kinetics.^[^ ^38^, ^39^, ^40^ ^]^ Considering the broad time window in the drying process of non‐halogenated solvents‐based high‐speed coated films, it is difficult to comprehensively consider these complex structural evolutions and optimize the BHJ morphology, let alone design feasible and widely applicable morphology optimization methods under varied manufacturing conditions. As continuous and environmentally friendly printing of photoactive layers become necessary for future production, these issues are expected to become more prominent.

In this work, utilizing a slot‐die processed PM6:BTP‐eC9^[^ ^41^, ^42^ ^]^ system, we demonstrated an effective donor‐priority rapid aggregation process (DP‐RAP) scheme during high‐speed coating for BHJ morphology optimization in non‐halogenated solvents‐based OSCs. The DP‐RAP scheme can effectively adjust the morphology and form a nanoscale fiber network structure by rational controlling the temperatures of solution and substrate. More importantly, this strategy can induce fibrous network morphology over a range of printing speeds and evaporation conditions (20–500 mm s^−1^), allowing the OSCs to maintain stable photovoltaic efficiency under fluctuating printing conditions. Detailed morphology and in situ ultraviolet–visible (UV–Vis) absorption measurements elucidated that the rapid aggregation process of donor components benefits the formation of polymer chain framework, which contributes to the formation of the fibrillar network structure. However, in the conventional slot‐die coating process, the acceptor aggregates shortly during the volatilization process, mainly resulted from the limited solubility of BTP‐eC9 in toluene, thus inducing the excessive acceptor crystallization and wrinkle‐type BHJ morphology. In addition, photoelectric characterizations show that the fibrillar morphology enables balanced carrier transport due to the reasonable crystallinity of donor and acceptor domains, while the wrinkle‐type morphology results in excessive electron mobility due the excessive acceptor crystallinity. Therefore, suppressed recombination is desired in the DP‐RAP based devices, contributing to a champion power conversion efficiency (PCE) of 17.4% at coting speed of 500 mm s^−1^ and without any further post‐treatment, which is one of the highest values reported for non‐halogenated solvent‐processed solar cells in the atmosphere. In particular, the DP‐RAP based devices processed at optimal temperatures remain a stable PCE of approximately 17.4% within the broad coating region of 20–500 mm s^−1^, illustrating the robustness and scalability of this strategy for high‐throughput manufacturing. This work provides a promising approach for high‐speed coating ecofriendly OSCs to achieve highly efficient and coating process tolerant devices, bringing them closer to eco‐compatible and practical manufacturing.

## 2 Results and Discussion

**Figure 1:**
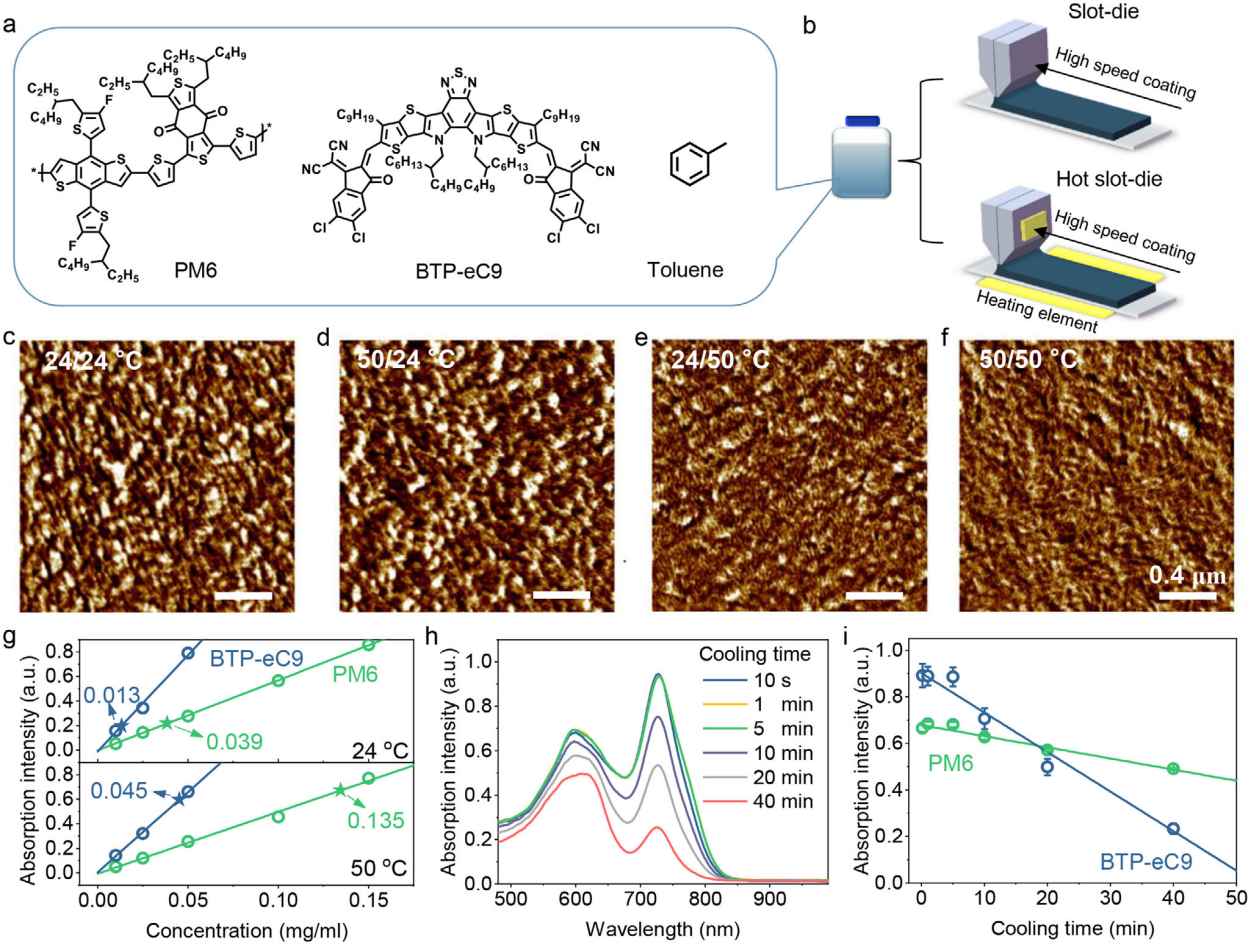
a) The chemical structure of PM6, BTP‐eC9, and toluene. b) Schematic diagram of high‐speed slot‐die coating and high‐speed hot slot‐die coating. c–f) The AFM phase images of PM6:BTP‐eC9 blends processed at c) 24/24 °C, d) 50/24 °C, e) 24/50 °C, and f) 50/50 °C, respectively. g) The concentration dependent absorption intensity extracted at 615 nm and 725 nm from the absorption spectra of PM6 and BTP‐eC9 toluene solutions. The green and blue stars indicate the concentration of the 100‐fold diluted saturated solution at corresponding temperatures. h) The cooling time dependent absorption spectra of PM6:BTP‐eC9 toluene solutions in custom chamber. i) Cooling time dependent absorption intensity extracted at 615 nm and 725 nm from the absorption spectra of PM6:BTP‐eC9 toluene solutions.

**Figure** **1**a shows the chemical structure of polymer donor poly[(2,6‐(4,8‐bis(5‐(2‐ethylhexyl‐3‐fluoro)thiophen‐2‐yl)‐benzo[1,2‐b:4,5‐b′]dithiophene))‐alt‐(5,5‐(1′,3′‐di‐2‐thienyl‐5′,7′‐bis(2‐ethylhexyl)benzo[1′,2′‐c:4′,5′‐c′]dithiophene‐4,8‐dione)] (PM6) and fused‐ring acceptor 2,2'‐[[12,13‐Bis(2‐butyloctyl)‐12,13‐dihydro‐3,9‐dinonylbisthieno[2″,3'':4′,5']thieno[2′,3':4,5]pyrrolo[3,2‐e:2′,3′‐g] [2,1,3]benzothiadiazole‐2,10‐diyl]bis[methylidyne(5,6‐chloro‐3‐oxo‐1H‐indene‐2,1(3H)‐diylidene)]]bis[propanedinitrile] (BTP‐eC9). A widely used high‐boiling point hydrocarbon solvent toluene was used to prepared PM6, BTP‐eC9, and their blend films. The neat and blend films were prepared by a typical slot‐die coating method with ultra‐high coating speed (over 450 mm s^−1^, details in Supporting Information). Heating elements were utilized to control the solution temperature (*T*_sol_) and substrate temperature (*T*_sub_), thereby regulating the solvent evaporation and aggregation kinetics of donor and acceptor (Figure 1b). To adjust the aggregation order and kinetics of donor and acceptor components and illustrate theirs impact on film morphology, PM6:BTP‐eC9 blends processed with diverse solution temperature and substrate temperature (24/24 °C, 50/24 °C, 24/50 °C, and 50/50 °C, the former value is the solution temperature and the later value is the substrate temperature) are investigated by atomic force microscopy (AFM) and transmission electron microscopy (TEM). As shown in Figure 1c–f and Figure S1 (Supporting Information), all the blends exhibit nanoscale domain distribution with small roughness (root‐mean‐square (RMS) values of 1.15, 1.16, 1.02, and 1.00 nm for 24/24 °C, 50/24 °C, 24/50 °C, and 50/50 °C films, respectively). Notably, the fibrillar type of morphology can be seen in the 24/50 °C and 50/50 °C blends, while wrinkle‐type morphology with no obvious fibrillar networks was observed in the 24/24 °C and 50/24 °C films.^[^ ^43^, ^44^, ^45^ ^]^ In addition, the TEM images (Figure S2, Supporting Information) indicate that the 24/24 °C and 50/24 °C blends show large‐scale phase separation, while the 24/50 °C and 50/50 °C blends show uniform and small‐scale phase separation.

To reveal the reasons for the morphological differences caused by solution/substrate temperature, the solubility of PM6 and BTP‐eC9 in toluene is quantified by UV–vis spectrophotometric analysis. The absorption spectra of PM6 and BTP‐eC9 solutions with various concentrations and at saturation state at room temperature (24 °C) and standard pressure (1 atmospheres) were measured and shown in Figure S3 (Supporting Information). The concentration dependent absorption intensity extracted at 615 and 725 nm from the absorption spectra of PM6 and BTP‐eC9 solutions were shown in Figure 1g. The absorption follows a proportional relationship with the solute concentrations, which is consistent with the Beer‐Lambert Law and can be used to determine the solute concentration at saturation, providing a direct way to quantify solubility. Here, the absorption spectra of a 100‐fold diluted saturated solution were measured to estimate the solubility of PM6 and BTP‐eC9 in toluene at different temperatures. Consequently, the solubility of PM6 in toluene at 24 °C and 50 °C can be quantified as 3.9 and 13.5 mg mL^−1^, respectively, and the solubility of BTP‐eC9 in toluene at 24 °C and 50 °C is 1.3 and 4.5 mg mL^−1^, respectively. The low solubility of BTP‐eC9 in toluene makes it easier to aggregate or even precipitate during film drying. Considering the strong self‐assembly ability of BTP‐eC9 as well as the slow evaporation of toluene, reasonable morphology control is crucial in high‐speed slot‐die printing.

The aggregation behavior of PM6 and BTP‐eC9 during film drying was further evaluated by UV–vis spectroscopy analysis. We are aware that the short‐range structural changes in solute aggregates and changes in solute concentration are able to change the interactions and concentration of chromophores.^[^ ^46^ ^]^ Based on the absorption changes in the UV–visible region, we can infer the spontaneous aggregation and precipitation of PM6 and BTP‐eC9 during film drying. Here, a spectrometer‐monitored liquid chamber was utilized to record the changes in absorption. This custom chamber with minimal air contract area to minimize toluene evaporation and maintain the solution concentration approximately constant (Figure S4, Supporting Information). Then, a hot supersaturated solution was transferred into the chamber to simulate the aggregation of solute during active layer deposition process. It is well known that the deposition of active layer is accompanied by the evaporation of solvent, which involves the supersaturation of the solution and subsequent plasticization of active layer. Our goal is to simulate the solvent evaporation process in film deposition by slow cooling process of a hot supersaturated solution, with the aid of liquid chamber, and then to infer the solute aggregation behavior in toluene coated films. Figure 1h shows the collected absorption spectrum. Within the first 10 s, two primary absorption peaks were observed at approximately 600 and 730 nm, corresponding to PM6 and BTP‐eC9, respectively. As cooling progressed, the absorption intensity decreased, and two PM6 peaks emerged at 615 nm (0–0 transition) and 575 nm (0–1 transition). This indicates the reduction in chromophore concentration and the enhanced molecular conjugation and backbone planarity.^[^ ^47^ ^]^ Finally, the initial broad peak at 600 nm gradually evolved to a more defined structure with main (615 nm) and shoulder (575 nm) peaks after 40 min, suggesting enhanced molecular ordering. Besides, the absorption intensity of BTP‐eC9 reduced significantly with time, and the peak location red‐shifted within 5 min, but blue‐shifted after 10 min (Figure S5, Supporting Information). This demonstrates that BTP‐eC9 aggregates within the first 5 minutes and subsequently precipitates, leading to a significant decrease in intensity and a blue shift of the peak after 10 minutes. The absorption intensity as a function of cooling time in the range of 570–620 nm and 715–745 nm extracted from the absorption spectra of PM6:BTP‐eC9 solutions were shown in Figure 1i. The rapid decrease of BTP‐eC9 absorption over time implies a faster aggregation and precipitation of BTP‐eC9 compared to PM6. These observation match well with the AFM results. Therefore, the nanoscale morphology difference of blends can be attributed to the aggregation order of PM6 and BTP‐eC9. The high substrate temperature is beneficial to maintaining a high solubility of BTP‐eC9 in toluene, thereby delaying the aggregation and precipitation of BTP‐eC9. Thus, PM6 can aggregate before BTP‐eC9 and form a polymer chain framework, laying the structural foundation for the fibrillar morphology.

**Figure 2:**
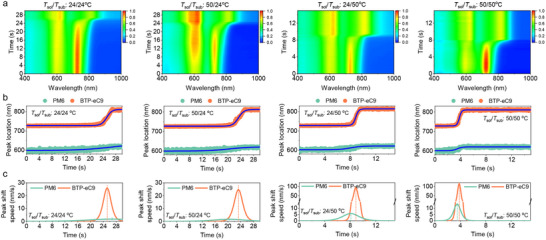
a) Time‐dependent contour maps of in situ UV–vis absorption and b) time‐dependent donor and acceptor peak location for PM6:BTP‐eC9 blends coated at 24/24 °C, 50/24 °C, 24/50 °C, and 50/50 °C at high coating speed over 450 mm s^−1^. c) Peak shift speed versus time of each blend obtained by plotting the derivative of the peak location over time. This derivative explains how fast the donor and acceptor peaks are shifting and can provide a quantitative view of the donor and acceptor aggregation process.

Further characterizations on the film drying process were done with in‐situ absorbance measurements to track the donor and acceptor aggregation kinetics. The time‐resolved UV–vis absorption spectra and related absorption contour maps are exhibited in Figure S6 (Supporting Information) and **Figure** **2**a. Figure 2b track the peak location evolution of PM6 and BTP‐eC9 from dissolved state to the solid state over time, suggesting a three‐stage film drying process. At first, the PM6 and BTP‐eC9 peaks in solution locate at around 600 nm and 725 nm, respectively (Stage I). Of note is that the long‐duration of this stage caused by the ultra‐high coating speed (Figure S7, Supporting Information). Therefore, fine control of solute aggregation in toluene is crucial for the development of non‐halogen solvents processed OSCs, especially for high‐speed coating. With solvent evaporation, the PM6 absorption peak red‐shifted to around 620 nm, while the BTP‐eC9 absorption peak red‐shifted to about 810 nm (Stage II). This suggests the aggregation of donors and acceptor molecules as well as the resultant ordered molecular packing.^[^ ^32^ ^]^ Finally, the absorption peak position remains unchanged, indicating the complete film solidification (Stage III). The peak location evolution of PM6 and BTP‐eC9 can be quantified via the Boltzmann function, and the fitting results are shown as blue curves in Figure 2b. The onset of stage II, the point where the absorption peak starts to shift, begins earlier (<4 s) for films coated at high substrate temperature, but slower (>12 s) for films coated at low substrate temperature. This primarily due to the faster evaporation rates of toluene, which can be attributed to the thermal conductivity from the hot substrate as well as the faster solution spreading rates.^[^ ^48^ ^]^

The absorption peak shift speeds can be evaluated according to the first derivative of the corresponding curves (Figure 2c), providing a closer view for the comparison of aggregation kinetics of PM6 and BTP‐eC9. Generally, a higher and narrower peak in Figure 2c represents a faster conversion rate, while a broader peak represents a slower molecular aggregation.^[^ ^10^ ^]^ Here, a higher peak shift rate corresponds to a shorter aggregation time, providing a useful metric for understanding the aggregation behavior of PM6 and BTP‐eC9. For example, in the 24/24 °C and 50/24 °C blends, PM6 aggregation occurs slowly, with a peak shift speed below 2 mm s^−1^. In contrast, the 24/50 °C and 50/50 °C blends exhibit faster PM6 aggregation, reaching maximum peak shift speed of approximately 6 and 13 mm s^−1^, respectively. This rapid aggregation in blends coated at high temperature suggests that PM6 can effectively overcomes the barrier and form locally ordered aggregates, which lays the structural foundation for fibrillar polymer networks. Meanwhile, all four films exhibit fast BTP‐eC9 aggregation, with maximum peak shift speed exceeding 25 mm s^−1^, and the highest value surpass 100 mm s^−1^ in the 50/50 °C blend. This suggests strong tendency of BTP‐eC9 for self‐assembly into ordered molecular packing. However, in the 24/50 °C and 50/50 °C blends, PM6 reaches its aggregation peak earlier than BTP‐eC9, confirming that PM6 aggregation precedes that of BTP‐eC9, while the heated substrate delays BTP‐eC9 aggregation. This donor‐priority rapid aggregation process (DP‐RAP) benefits the formation of a fibrillar network structure. Considering the AFM results, it's reasonable to conclude that sufficient PM6 chain networks and the subsequent aggregation of BTP‐eC9, are the main factors driving the formation of fibrillar morphology in the 24/50 °C and 50/50 °C blends. In contrast, for 24/24 °C and 50/24 °C films, the slow aggregation of PM6 makes it difficult to form the framework of ordered polymer chains, even if PM6 aggregates preferentially over BTP‐eC9 in the 50/24 °C film. Consequently, the DP‐RAP strategy is beneficial for the confined aggregation of BTP‐eC9 in the preferential formed polymer network, which thus benefits the development of nanoscale fiber morphology.

**Figure 3:**
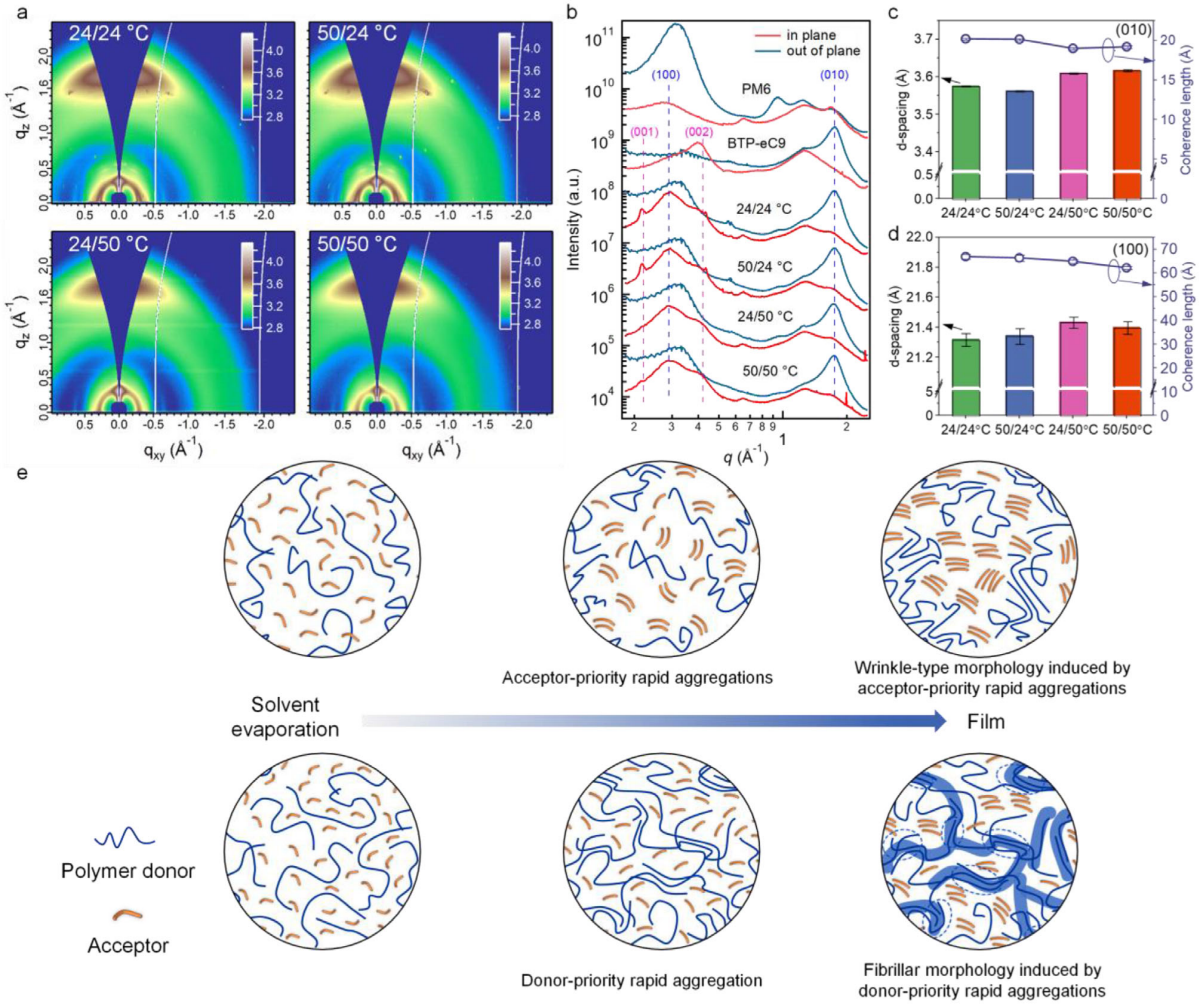
a) 2D GIWAXS patterns of the PM6:BTP‐eC9 blends coated at 24/24 °C, 50/24 °C, 24/50 °C, and 50/50 °C with high coating speed over 450 mm s^−1^. b) GIWAXS line profiles of the neat and blend films. c,d) The d‐spacing and coherence length of c) peak (100) and d) (010) for the blend films. e) Schematic illustration of the distinction stages within the film drying process for the selected 24/24 °C and 50/50 °C films. The acceptor‐priority rapid aggregation in 24/24 °C film can induce excessive acceptor crystallization and the wrinkle‐type morphology, while the donor‐priority rapid aggregation in 50/50 °C film can contribute to polymer chains framework and the fibrillar morphology.

Further morphological characterizations were done with grazing‐incidence wide‐angle X‐ray scattering (GIWAXS) to interpret the effect of PM6 and BTP‐eC9 aggregation kinetics on the molecular ordering.^[^ ^49^ ^]^ The 2D GIWAXS patterns and line curves of the neat and blend films are shown in Figure S7 (Supporting Information) and **Figure** **3**, respectively. All the blends along with the neat BTP‐eC9 film exhibit face‐on dominated orientation with a broadened (010) scattering peak of π‐π stacking in the out‐of‐plane (OOP) direction, while PM6 film shows a prominent lamellar (100) scattering peak at 0.31 Å^−1^ along the OOP direction, suggesting the edge‐on dominated orientation. The OOP scattering peaks at about 0.31 Å^−1^ and the in‐plane (IP) scattering peaks at about 0.30 Å^−1^ in PM6:BTP‐eC9 blends can be assigned to the PM6 lamellar stacking, while the IP scattering peaks at about 0.22 and 0.40 Å^−1^ mainly results from the BTP‐eC9 (001) and (002) packing, respectively.^[^ ^50^ ^]^ Of note is that the BTP‐eC9 (001) peak is only presence in 24/24 °C and 50/24 °C blends, this indicates the long‐range ordered backbone packing of BTP‐eC9. Considering to the in situ absorbance results, it is reasonable to consider that the long‐range BTP‐eC9 backbone packing originates from the smaller PM6 spatial confinement. As a comparison, the disappear of BTP‐eC9 (001) peak in 24/50 °C and 50/50 °C blends suggests less ordered backbone packing, particularly due to the spatial constraints of the polymer chain framework and the rapid acceptor aggregation process. The molecular ordering was further evaluated via the fitting of those curves and the Scherrer analysis.^[^ ^51^ ^]^ As shown in Figure 3c,d and Table S1 (Supporting Information), the d‐spacing (coherence length, *L*_C_) of (010) π–π stacking in 24/24 °C, 50/24 °C, 24/50 °C, and 50/50 °C blends are 3.57 ± 0.001 Å (20.19 ± 0.11 Å), 3.56 ± 0.0001 Å (20.13 ± 0.10 Å), 3.61 ± 0.002 Å (18.97 ± 0.11 Å), and 3.62 ± 0.002 Å (19.18 ± 0.15 Å), respectively. Thus, the *g*‐factors of π‐π stacking are estimated to be 15.9%, 15.9%, 16.5%, and 16.4%, respectively. Moreover, the *L*_C_ of PM6 lamellar packing in 24/24 °C and 50/24 °C blends are higher than those in 24/50 °C and 50/50 °C blends (Table S2, Supporting Information). These results suggest higher molecular crystallinity of donor and acceptor components in the 24/24 °C and 50/24 °C blends.

Based on the above results, a complete picture of the morphological evolution in the PM6:BTP‐eC9 blends coated at different solvent and substrate temperatures can be deduced. Take the 24/24 °C and 50/50 °C blends as example, the impact of temperature on donor and acceptor aggregation kinetics and the subsequent film morphology is illustrated in Figure 3e,f. PM6 is well soluble in toluene at 24 °C and 50 °C and exhibits loose solution aggregation in hot solutions due to its temperature‐dependent aggregation properties, and BTP‐eC9 shows low aggregation tendency in warm toluene solution due to the increased solubility. With the evaporation of toluene, BTP‐eC9 in the 24/24 °C blend aggregates early due to its limited solubility in toluene, and then PM6 aggregates slowly under the spatial confinement of the aggregated acceptor domains. In contrast, the 50/50 °C sample experiences PM6‐priority chain aggregation due to the thermal delayed acceptor aggregation. It should be noted that the early aggregation of PM6 would yields ordered donor chain locally due to the rapid toluene evaporation that enhances the aggregation kinetics. The PM6 aggregates then assemble into fibrillar structures that lay the foundation of frameworks for the subsequent aggregation of BTP‐eC9, which thus contributes to the formation of the nanoscale fibrillar morphology. Therefore, the 50/50 °C sample exhibits a fibrillar type morphology induced by donor‐priority aggregation, while the 24/24 °C sample shows a wrinkled morphology induced by the acceptor‐priority aggregation. In addition, the slower evaporation of toluene facilitates stronger donor and acceptor crystallization in 24/24 °C blends. As a result, the hot slot‐die strategy can effectively optimize the nanoscale fibrillar morphology by tuning the aggregation order and kinetics of both donor and acceptor components in ultra‐high‐speed toluene‐processed blends, which further lays a good morphological foundation for efficient charge transport and photovoltaic performance.

**Figure 4:**
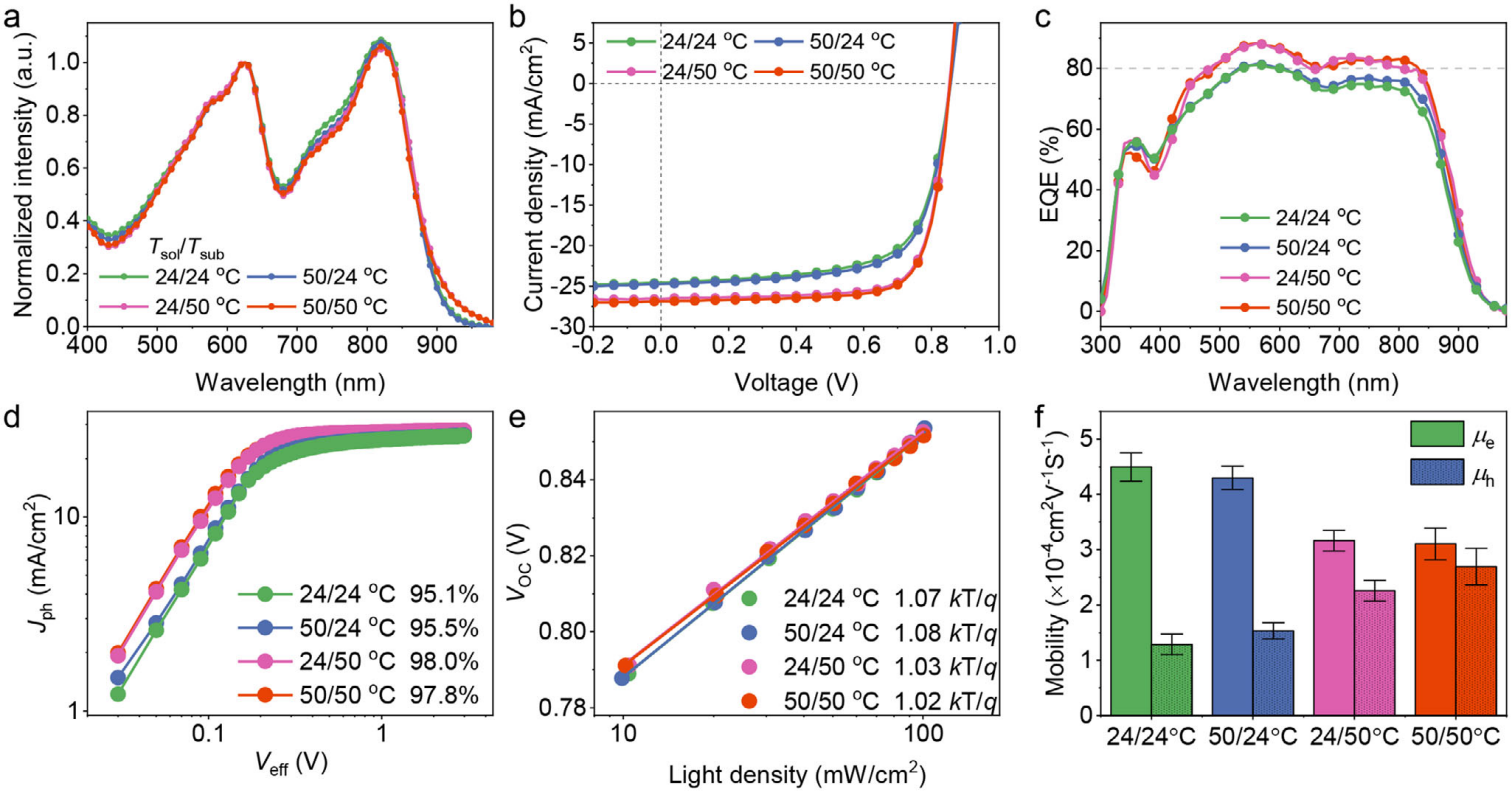
a) Normalized absorption spectra, b) current density–voltage (*J*–*V*) characteristic curves and c) EQE response curves of PM6:BTP‐eC9 based devices fabricated at 24/24 °C, 50/24 °C, 24/50 °C, and 50/50 °C with high coating speed over 450 mm s^−1^. d) *J*_ph_–*V*_eff_ curves, e) light intensity dependent *V*_OC_ curves and (f) carrier mobility of the PM6:BTP‐eC9 based devices coated at different conditions.

Further characterization on the blend absorption was performed with the UV‐Vis absorption spectroscopy to explore the dependence of light absorption on blend morphology, The normalized absorption spectra of PM6:BTP‐eC9 blends with a controlled thickness of 110 nm are shown in **Figure** **4**a. The BTP‐eC9 absorption in 24/24 °C and 50/24 °C blends is higher than that in 24/50 °C and 50/50 °C blends. Furthermore, the ratio of BTP‐eC9 0‐0 peak to 0–1 peak was decreased in the 24/24 °C and 50/24 °C blends compared to the 24/50 °C and 50/50 °C blends. These results correlate well with the decreased crystallinity 24/50 °C and 50/50 °C blends.

Conventional OSCs with the device architecture of indium tin oxide (ITO)/PEDOT:PSS/PM6:BTP‐eC9/PDINO/Al were fabricated to investigate the relations between the active layer morphology and photovoltaic parameters. The detailed conditions, including the solution concentration, D:A ratio and coating speed are summarized in the Supporting Information. The coating temperature‐dependent device parameters including open‐circuit voltage (*V*_OC_), short‐circuit current density (*J*_SC_), fill factor (FF) and PCE are summarized in **Table** **1**, and the current density versus voltage (*J*–*V*) curves of the champion devices are plotted in Figure 4b. Devices fabricated at 24/24 °C exhibited lower PCE (14.1 ± 0.3%) at coating speed of 450 mm s^−1^. However, increasing the solution temperature (50/24 °C) only lead to a slight increase of PCE to 14.7 ± 0.5% at coating speed of 500 mm s^−1^. In contrast, increasing the substrate temperature to 50 °C, devices showed improved *J*_SC_ and FF. This contributes to the enhanced PCE of 17.0 ± 0.4% at coating speed of 450 mm s^−1^ and highlights the benefits of hot substrate in high‐speed slot‐die coating. Furthermore, devices fabricated at 50/50 °C showed improved *J*_SC_ and FF, yielding the enhanced PCE of 17.1 ± 0.3%. Particularly, a champion PCE of 17.4% was achieved at coating speed of 500 mm s^−1^, which is among the highest values reported for non‐halogenated solvent processed devices under high‐speed conditions.^[^ ^7^, ^23^ ^]^

**Table 1:** Photovoltaic parameters of PM6:BTP‐eC9 based OSCs under the illumination of AM 1.5G, 100 mW cm^−2^ (The average values are obtained from at least 10 devices).

*T*_sol_/*T*_Sub_	Coating speed [mm s^−1^]	*V*_OC_ [V]	*J*_SC_ [mA cm^−2^]	FF [%]	PCE [%]
24/24 °C	450	0.856 (0.854 ± 0.006)	24.6 (24.2 ± 0.7)	67.9 (66.7 ± 1.6)	14.3 (14.1 ± 0.3)
50/24 °C	500	0.857 (0.855 ± 0.005)	24.8 (24.3 ± 0.8)	69.3 (68.5 ± 1.8)	14.7 (14.4 ± 0.5)
24/50 °C	450	0.854 (0.853 ± 0.004)	26.6 (26.3 ± 0.4)	76.1 (75.7 ± 1.2)	17.3 (17.0 ± 0.4)
50/50 °C	500	0.855 (0.855 ± 0.003)	26.9 (27.0 ± 0.3)	75.9 (75.2 ± 0.8)	17.4 (17.1 ± 0.3)

External quantum efficiency (EQE) measurements were performed to determine the *J*_SC_. As shown in Figure 4c, the integrated current of 24/24 °C, 50/24 °C, 24/50 °C, and 50/50 °C devices was 24.41, 23.90 26.31, and 26.44 mA cm^−2^, respectively, which are in good agreement with the *J*_SC_ values obtained from the *J*–*V* measurements. Particularly, the EQE response of 24/50 °C and 50/50 °C devices are higher than that of 24/24 °C and 50/24 °C across the 450–850 nm region, suggesting sufficient charge generation. Moreover, for 24/50 °C and 50/50 °C devices, the improved charge dissociation and collection efficiency estimated by bias voltage‐dependent photocurrent is also responsible for the high *J*_SC_. As shown in Figure 4d, the charge dissociation probability (*P*_d_) can be evaluated by the ratio of *J*_ph_/*J*_sat_ under short‐circuit conditions (*J*_ph_ is photocurrent density and *J*_sat_ is the saturation photocurrent density).^[^ ^52^, ^53^ ^]^ The 24/50 °C and 50/50 °C solar cells exhibited *P*_d_ values of 98.0% and 97.8%, respectively, demonstrating better charge dissociation probabilities.

The dependence of *J*_SC_ and *V*_OC_ on the light intensity was examined to explore the charge recombination in these devices. Generally, the *V*_OC_(*P*) relation is used to distinguish whether Shockley‐Read‐Hall (SRH) recombination or bimolecular recombination dominates the carrier recombination process (via the slop of *kT*/*q*, where *k* is the Boltzmann constant and *T* is the absolute temperature).^[^ ^54^, ^55^ ^]^ As shown in Figure 4e, the slopes of 24/50 °C (1.03 *kT*/*q*) and 50/50 °C (1.02 *kT*/*q*) devices are smaller than those of 24/24 °C (1.07 *kT*/*q*) and 50/24 °C (1.08 *kT*/*q*) devices, suggesting low SRH recombination in the DP‐RAP‐based devices. Quantitatively, the bimolecular recombination loss under short‐circuit condition is characterized by the *J*_SC_(*P* ^α^) model, where *α* is a power‐law exponent extracted from the slope of the data on a log‐log scale. As shown in Figure S9 (Supporting Information), devices fabricated at 24/50 °C and 50/50 °C exhibited α values of 0.991 and 0.993, respectively. The values close to 1 indicate the weak bimolecular recombination losses than other counterparts. The hole and electron mobility were measured to further understand the change of FF via space charge limited current (SCLC) model.^[^ ^56^ ^]^ The *J*–*V* curves of the hole‐only and electron‐only devices were shown in Figure S10 (Supporting Information), and the calculated hole mobility (*µ*_h_) and electron mobility (*µ*_e_) of relative devices were summarized in Figure 4f and Table S3 (Supporting Information). The averaged *µ*_h_ values of the 24/24 °C, 50/24 °C, 24/50 °C, and 50/50 °C devices were 1.29×10^−4^, 1.53×10^−4^, 2.26×10^−4^, and 2.70×10^−4^ cm^2^ V^−1^ S^−1^, respectively, while the *µ*_e_ values for these devices were 4.49×10^−4^, 4.30×10^−4^, 3.16×10^−4^, and 3.10×10^−4^ cm^2^ V^−1^ S^−1^, respectively. Therefore, a more balanced *µ*_h_/*µ*_e_ ratio were observed in the 50/50 °C devices, which yields reduced space‐charge accumulation and subsequent high FF in the devices. In summary, the improved carrier dynamics behaviors strongly underpin the molecular crystallinity and phase‐separated morphology in the DP‐RAP‐based devices, and can well explain their superior photovoltaic parameters.

**Figure 5:**
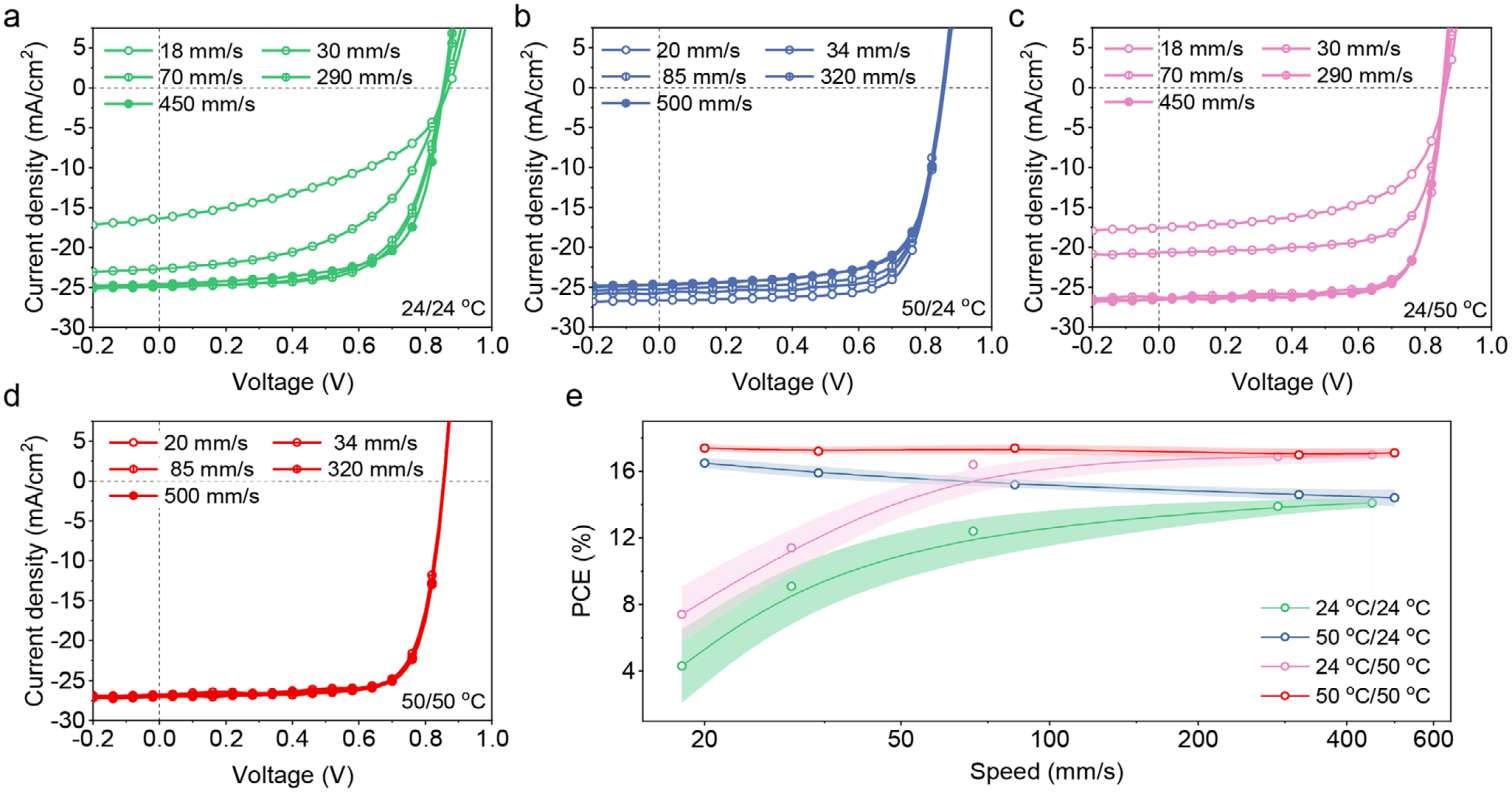
a–d) The typical *J*–*V* curves and e) averaged PCEs of PM6:BTP‐eC9 based devices fabricated at 24/24 °C, 50/24 °C, 24/50 °C, and 50/50 °C with various coating speeds.

The non‐halogenated solvents are typically considered for scale‐up production of OSCs due to their low toxicity. Compared to the typical coating process using chloroform, the slot‐die coating using non‐halogenated solvents shows advantages in continuous fabrication of OSCs. Therefore, we further investigated the applicability of DP‐RAP strategy in continuous fabrication under various coating speed. The typical *J–V* curves of OSCs coated at various conditions are shown in **Figure** **5**a–d, with detailed photovoltaic parameters shown in Tables S4–S7 (Supporting Information). With the decrease of coating speed, the 24/24 °C‐based and 24/50 °C‐based OSCs shows decreased PCE (4.3 ± 2.2% and 7.4 ± 1.7% at 18 mm s^−1^, respectively). However, the 50/24 °C‐based devices achieved gradually increased PCE up to 16.5 ± 0.3% at 18 mm s^−1^, compared to the device efficiency coated at ultra‐high coating speed of 500 mm s^−1^ (14.7 ± 0.5%). In contrast, the 50/50 °C‐based devices showed almost unchanged photovoltaic performance across the wide coating speed window of 20–500 mm s^−1^. The PCE values for these devices coated at various conditions are summarized in Figure 5e, suggesting the extremely low performance fluctuations of the 50/50 °C‐based devices at various coating speeds.

To further investigate the variations in device performance, we conducted photoelectrical and morphological characterizations. Figure S11 (Supporting Information) shows the UV–Vis absorption spectra and EQE response of devices processed at low‐coating speeds. The low‐speed coated 24/24  °C and 24/50  °C blends exhibit a significant reduction in absorption intensity within the 650–900 nm range, which is attributed to the precipitation of BTP‐eC9. Given the low solubility of BTP‐eC9 in toluene at 24 °C (1.3 mg mL^−1^), this precipitation reduces BTP‐eC9's contribution to photocurrent generation, leading to a significant drop in EQE and lower *J*_SC_ in related devices. In contrast, the low‐speed coated 50/24  °C device demonstrates higher EQE responses than its high‐speed coated counterpart. This improvement can be attributed to the rapid film‐drying process at low coating speeds, which prevents excessive BTP‐eC9 aggregation and phase separation. Notably, devices coated at 50/50  °C exhibit similar absorption and EQE responses at both high and low speeds, leading to stable performance across a broad coating speed range. The morphology of low‐speed coated blends was further analyzed by using TEM and AFM (Figure S12, Supporting Information). The AFM and TEM phase images reveal that the low‐speed coated 24/24 °C and 24/50  °C blends exhibit large‐scale phase separation and a wrinkle‐type morphology, which contributes to the lower FF observed in these devices. In contrast, the low‐speed coated 50/24  °C blend exhibits a fibrillar‐type morphology with a smaller phase separation scale, explaining its improved FF compared to high‐speed coated devices. Moreover, blends coated at 50/50  °C display similar phase separation scales and fiber network structures with low surface roughness. These findings suggest that the DP‐RAP strategy remains effective at low coating speeds by maintaining a stable fibrillar morphology, ensuring high tolerance to coating speed the variations in the continuous fabrication of OSC using non‐halogenated solvents.

## 3 Conclusion

In summary, we developed an effective DP‐RAP strategy that achieved an improved morphology and higher device performance in the ultrahigh‐speed slot‐die coating process with eco‐friendly solvent. An in‐depth analysis of aggregation kinetics and morphology variations revealed that a donor‐priority aggregation process is beneficial for the nanoscale fiber network structure to avoid severe small molecular aggregation. In addition, the DP‐RAP scheme achieved at specific solution temperature and substrate temperature exhibited an accelerated evaporation rate, which shortened the phase separation process and favored the formation of uniform and small‐scale phase separation. Both advantages facilitated the formation of a fibrillar network morphology with reasonable donor and acceptor domains in the active layer. Therefore, the resultant toluene‐processed OSCs exhibited a champion PCE of 17.4% at coating speed of 500 mm s^−1^ and without any further post‐treatment, which is one of the highest values reported for eco‐friendly solar cells in the atmosphere. In addition, the DP‐RAP strategy shown great tolerance to the coating speed fluctuations with remaining a stable PCE of approximately 17.4% within the broad coating region of 20–500 mm s^−1^. These outcomes provide a new approach for achieving optimal blend morphology in high‐speed slot‐die processing, which was shown to be a practical and promising strategy for the high‐throughput fabrication of efficient and eco‐compatible OSCs.

## Conflict of Interest

The authors declare no conflict of interest.

## Supporting information



Supporting Information

Supporting Data

## Data Availability

The data that support the findings of this study are available from the corresponding author upon reasonable request.
