# Supplementary material for: High‐Speed Slot‐Die Coating with Donor‐Priority Rapid Aggregation Kinetics for Improved Morphology and Efficiency in Ecofriendly Organic Solar Cells
Source: Adv Sci (Weinh). 2025 Apr 26;12(27):2502077. doi: 10.1002/advs.202502077 (PMC12279230; doi:10.1002/advs.202502077)
Supplement: Supplementary file 1 — Supporting Information [file ADVS-12-2502077-s002.docx]

Supporting Information

High-Speed Slot-Die Coating with Donor-Priority Rapid Aggregation Kinetics for Improved Morphology and Efficiency in Ecofriendly Organic Solar Cells

Zhaozhao Bi, Baohua Wu, Ke Wang, Jingwei Xue, Chang Liu, Lingxiao Tang, Ke Zhou*, Long Jiang*, and Wei Ma*

# Supporting Methods

Materials

PM6 and BTP-eC9 were purchased from Solarmer Materials Inc. (China). The solvent toluene was purchased from Sinopharm Chemical Reagent Co., Ltd (China). PEDOT:PSS(Al 4083) used to prepare solar cell devices, and AFM samples was purchased from Heraeus. The glass/ITO substrates were purchased from South China Science & Technology Co., Ltd.

Device fabrication

The organic solar cells were fabricated with a conventional architecture of indium tin oxide (ITO)/PEDOT:PSS/Active layer/PDINO/Al. The patterned ITO substrate was continuously cleaned three times by sonication in water with detergent, deionized water, acetone, and isopropanol for 30 min of each step. Then the substrate was dried with a nitrogen gun. After ultraviolet ozone treatment for 20 min, the PEDOT:PSS layer was spin-coated at 5500 rpm onto the ITO glass substrates as hole transporting layer with a thickness of 30 nm, followed by annealing at 150 °C for 15 min. The active layer solution was prepared in toluene with different total concentrations (D/A = 1:1.2 by weight) without any additives. After that, a 100 nm film of the active layer was coated by slot-die coating in ambient conditions. For the slot-die coating, the solution was pumped onto a moving substrate through the slot in the coating head. The gap between the substrate and the head was 100 μm. The slot-die head and substrate temperature were controlled by heating element. The coating speed of the substrate and the injection speed of the solution were adjusted according to different combination of solution concentration and coating temperature. Then the deposited films were transferred to the N_2_-ﬁlled glovebox without further treatment. Afterwards, a 10 nm PDINO was spin-coated at 3300 rpm onto the active layers. Finally, 100 nm Al were deposited as anode below the vacuum level of 1×10^−4^ Pa.

Morphology characterization

GIWAXS measurements were performed at beamline 7.3.3 at the Advanced Light Source.^[1]^ Samples were prepared on Si substrates using identical blend solutions as those used in devices. The 10 keV X-ray beam was incident at a grazing angle of 0.11°-0.15°, selected to maximize the scattering intensity from the samples. The scattered x-rays were detected using a Dectris Pilatus 2M photon counting detector. TEM characterization was performed by a FEI Talos F200c transmission electron microscope at 200 kV. AFM images of the active layers were obtained using Veeco INNOVA Atomic Force Microscope operated at tapping mode.

Optical characterization

Static UV-vis absorption spectra were acquired on Shimadzu UV-3600 Plus Spectrophotometer. The *in-situ* UV-vis absorption measurement was performed by

the Filmetrics F20-EXR spectrometer using the transmission mode with the time resolution of 0.02 s. The spectrometer consists of light source and detector. The light source and detector are fixed above and below the substrate, respectively, and on the same vertical line. The solution was injected into the slot and the film was coated onto the glass substrate. The detector collects the transmission spectra ranged from 400 to 1050 nm during coating. The UV-vis absorption spectra are calculated from the transmission spectra according to the equation A_λ_ = -log_10_(T), where A_λ_ is the absorbance at a certain wavelength (λ) and T is the calculated transmittance. The light source and detector were turned on before coating the film, so time zero is the point when the first solution transmission spectrum was collected by the detector. Before time zero, there is only noise in the transmission spectra.

Photovoltaic characterization

The *J-V* characteristics were measured by using an AAA solar simulator (SS-F5-3A, Enli Technology CO., Ltd.) in the N_2_ glove box. The radiative intensity (AM 1.5G spectrum, 100 mW cm-2) was calibrated by a standard silicon cell with a KG5 filter and a Keighley 2400 source meter unit. Typical devices area (0.04 cm^2^) was defined by a metal mask with aligned aperture. The EQE response were measured by a solar cell spectral response measurement system (QE-R3018, Enli Technology CO., Ltd.) with the calibrated light intensity by a standard single-crystal Si photovoltaic cell.

Carrier mobility measurement

The carrier mobilities were measured using the space charge limited current (SCLC) method, employing a hole-only device structure (ITO/PEDOT:PSS/Active layer/MoO_x_/Al) and an electron-only device structure (ITO/ZnO/Active layer/PDINO/Al). The mobilities were obtained by taking current density-voltage curves and fitting the results to a SCLC form, where the SCLC is described as:

*J* = 9*ε_0_ε*_r_*μ(V_appl_ - V_bi_* *- V_s_)*^2^*/*8*L*^3^

Where *J* is current density, *ε_0_* is the permittivity of free space, *ε*_r_ is the relative permittivity of the material (assumed to be 3), *μ* is hole mobility or electron mobility, *V_appl_* is applied voltage, *V_bi_* is the built-in voltage, *V_s_* is the voltage drop from the substrate’s series resistance (*V_s_*=IR) and L is the thickness of film.

# Supplementary Figures

Figure S1. The AFM height images of PM6:BTP-eC9 blends processed at 24/24 ^o^C, 50/24 ^o^C, 24/50 ^o^C, and 50/50 ^o^C, respectively.

Figure S2. The TEM images of PM6:BTP-eC9 blends processed at 24/24 ^o^C, 50/24 ^o^C, 24/50 ^o^C, and 50/50 ^o^C, respectively.

Figure S3. The absorption spectra of a series of PM6 and BTP-eC9 concentrations in toluene at 24 °C (a) and 50 °C (b).

Figure S4. Schematic diagram of the custom chamber and absorption spectrum testing process to infer the spontaneous aggregation and precipitation of PM6 and BTP-eC9 in PM6:BTP-eC9 toluene solution.

Here, the liquid chamber consists of two stacked transparent optical glass plates. During the experiment, heated solution was first transferred into the chamber (Figure S4I). Subsequently, the glass plates were aligned to minimize the air-liquid interface (Figure S4II), which significantly reduced solvent evaporation. This allowed us to observe the natural cooling process of the solution, simulating the solute aggregation behavior during film deposition. The liquid chamber was then placed on the spectrometer stage, which was maintained at 24 ^o^C to ensure a controlled cooling environment. By continuously monitoring the absorption spectra during this process, we could track solute aggregation and precipitation (Figure S4III). This setup enables us to analyze the spontaneous aggregation and phase behavior of PM6 and BTP-eC9 in toluene solutions under controlled cooling conditions.

Figure S5. The cooling time-dependent absorption changes of BTP-eC9 in PM6:BTP-eC9 toluene solution.

**Figure S6**. Time-resolved UV-vis absorption spectra for PM6:BTP-eC9 blends coated at 24/24 ^o^C, 50/24 ^o^C, 24/50 ^o^C, and 50/50 ^o^C, respectively. The absorption spectrum is calculated by transmission spectrum, with a formula of *A*(λ)=-lg(*T*(λ)), where λ is the wavelength, *A*(λ) is the absorbance, and *T*(λ) is the measured transmittance^[2]^.

**Figure S7**. (a) Film thickness measured as a function of coating speed in slot-die coating. Insert is the schematic diagram of slot-die coating. (b) Schematic diagram of coating speed induced liquid film and film formation mechanism.

**Figure S8**. Two-dimensional GIWAXS patterns of PM6 and BTP-eC9 neat films.

**Figure S9**. Light density dependent photocurrent curves of PM6:BTP-eC9 based devices processed at 24/24 ^o^C, 50/24 ^o^C, 24/50 ^o^C, and 50/50 ^o^C, respectively.

**Figure S10**. Hole (a) and electron (b) mobility of PM6:BTP-eC9 based devices processed at 24/24 ^o^C, 50/24 ^o^C, 24/50 ^o^C, and 50/50 ^o^C, respectively.

**Figure S11**. The absorption spectra of PM6:BTP-eC9 blends (a-d) and the EQE response curves of PM6:BTP-eC9 devices (e-h) processed at 24/24 ^o^C, 50/24 ^o^C, 24/50 ^o^C and 50/50 ^o^C under different coating speeds.

**Figure S12**. The AFM phase images (a) and TEM images (b) of PM6:BTP-eC9 blends processed at different temperatures under low-coating speed of 18-20 mm/s.

# Supplementary Tables

**Table S1**. GIWAXS fitting results of (010) scattering peaks of PM6:BTP-eC9 based blends.

| PM6:BTP-eC9 | q/Å^-1^ | d-spacing/Å | CL/Å | g (%) |
| --- | --- | --- | --- | --- |
| 24/24 ^o^C | 1.76 | 3.57 | 20.19 | 15.9 |
| 50/24 ^o^C | 1.76 | 3.56 | 20.13 | 15.9 |
| 24/50 ^o^C | 1.74 | 3.61 | 18.97 | 16.5 |
| 50/50 ^o^C | 1.74 | 3.62 | 19.18 | 16.4 |

The paracrystalline disorder (g-parameter) along the π-stacking direction (010) was obtained by the center position (*q*_y_) and FWHM of diffraction peak.^[3]^

**Table S2**. GIWAXS fitting results of (100) scattering peaks of PM6:BTP-eC9 based blends.

| PM6:BTP-eC9 | q/Å^-1^ | d-spacing/Å | CL/Å | g (%) |
| --- | --- | --- | --- | --- |
| 24/24 ^o^C | 0.29 | 21.32 | 66.85 | 21.4 |
| 50/24 ^o^C | 0.29 | 21.34 | 66.30 | 21.5 |
| 24/50 ^o^C | 0.29 | 21.43 | 64.88 | 21.8 |
| 50/50 ^o^C | 0.29 | 21.40 | 62.16 | 22.2 |

**Table S3**. The calculated carrier mobility results of PM6:BTP-eC9 based devices processed at 24/24 ^o^C, 50/24 ^o^C, 24/50 ^o^C, and 50/50 ^o^C, respectively.

| PM6:BTP-eC9 | Hole mobility  (×10^-4^ cm^2^V^-1^S^-1^) | Electron mobility  (×10^-4^ cm^2^V^-1^S^-1^) |
| --- | --- | --- |
| 24/24 ^o^C | 1.29±0.18 | 4.50±0.26 |
| 50/24 ^o^C | 1.53±0.15 | 4.30±0.21 |
| 24/50 ^o^C | 2.26±0.19 | 3.16±0.19 |
| 50/50 ^o^C | 2.70±0.32 | 3.10±0.28 |

**Table S4**. Photovoltaic parameters of PM6:BTP-eC9 based OSCs processed at 24/24 ^o^C under the illumination of AM 1.5G, 100 mW/cm^2^ (The average valuess are obtained from at least 10 devices).

| Coating speed (mm/s) | *V*_OC_ (V) | *J*_SC_ (mA/cm^2^) | FF (%) | PCE (%) |
| --- | --- | --- | --- | --- |
| 18 | 0.870  (0.878±0.008) | 16.3  (11.5±5.4) | 44.0  (41.9±3.2) | 6.3  (4.3±2.2) |
| 30 | 0.859  (0.864±0.012) | 22.7  (20.1±2.8) | 53.1  (50.7±2.4) | 10.3  (9.1±1.6) |
| 70 | 0.854  (0.851±0.006) | 24.9  (23.7±1.2) | 64.3  (63.1±1.2) | 13.7  (12.4±1.3) |
| 290 | 0.858  (0.855±0.003) | 24.8  (24.3±0.5) | 66.1  (65.2±0.9) | 14.1  (13.9±0.4) |
| 450 | 0.856  (0.854±0.006) | 24.6  (24.2±0.7) | 67.9  (66.7±1.6) | 14.3  (14.1±0.3) |

**Table S5**. Photovoltaic parameters of PM6:BTP-eC9 based OSCs processed at 50/24 ^o^C under the illumination of AM 1.5G, 100 mW/cm^2^ (The average valuess are obtained from at least 10 devices).

| Coating speed (mm/s) | *V*_OC_ (V) | *J*_SC_ (mA/cm^2^) | FF (%) | PCE (%) |
| --- | --- | --- | --- | --- |
| 20 | 0.852  (0.849±0.003) | 26.7  (26.3±0.5) | 74.1  (73.5±1.3) | 16.9  (16.5±0.4) |
| 34 | 0.855  (0.852±0.006) | 25.7  (25.4±0.4) | 73.2  (72.9±0.9) | 16.1  (15.9±0.4) |
| 85 | 0.852  (0.851±0.004) | 25.3  (24.9±0.5) | 72.7  (72.3±0.6) | 15.6  (15.2±0.4) |
| 320 | 0.854  (0.853±0.002) | 24.6  (24.3±0.3) | 70.8  (70.3±1.4) | 14.9  (14.6±0.3) |
| 500 | 0.857  (0.855±0.005) | 24.8  (24.2±0.7) | 69.3  (68.5±1.8) | 14.7  (14.4±0.5) |

**Table S6**. Photovoltaic parameters of PM6:BTP-eC9 based OSCs processed at 24/50 ^o^C under the illumination of AM 1.5G, 100 mW/cm^2^ (The average valuess are obtained from at least 10 devices).

| Coating speed (mm/s) | *V*_OC_ (V) | *J*_SC_ (mA/cm^2^) | FF (%) | PCE (%) |
| --- | --- | --- | --- | --- |
| 18 | 0.864  (0.873±0.012) | 17.6  (15.1±2.4) | 59.2  (55.6±4.7) | 9.0  (7.4±1.7) |
| 30 | 0.861  (0.859±0.009) | 20.7  (18.8±1.9) | 71.4  (69.3±2.8) | 12.7  (11.4±1.3) |
| 70 | 0.859  (0.855±0.004) | 26.3  (25.8±0.6) | 75.1  (74.6±1.1) | 16.9  (16.4±0.6) |
| 290 | 0.855  (0.852±0.003) | 26.5  (26.2±0.3) | 75.5  (75.2±0.8) | 17.2  (16.9±0.5) |
| 450 | 0.854  (0.853±0.004) | 26.6  (26.3±0.4) | 76.1  (75.7±1.2) | 17.3  (17.0±0.4) |

**Table S7**. Photovoltaic parameters of PM6:BTP-eC9 based OSCs processed at 50/50 ^o^C under the illumination of AM 1.5G, 100 mW/cm^2^ (The average valuess are obtained from at least 10 devices).

| Coating speed (mm/s) | *V*_OC_ (V) | *J*_SC_ (mA/cm^2^) | FF (%) | PCE (%) |
| --- | --- | --- | --- | --- |
| 20 | 0.855  (0.853±0.002) | 26.9  (27.1±0.2) | 76.6  (75.5±1.5) | 17.6  (17.4±0.2) |
| 34 | 0.857  (0.854±0.004) | 26.8  (26.8±0.4) | 76.3  (75.7±1.3) | 17.5  (17.2±0.5) |
| 85 | 0.855  (0.852±0.005) | 27.0  (27.2±0.7) | 76.0  (75.0±1.9) | 17.6  (17.4±0.3) |
| 320 | 0.855  (0.855±0.002) | 26.9  (26.8±0.9) | 75.4  (76.1±1.0) | 17.3  (17.0±0.4) |
| 500 | 0.855  (0.855±0.003) | 26.9  (27.0±0.3) | 75.9  (75.2±0.8) | 17.4  (17.1±0.3) |

**Supplementary References**

[1] A. Hexemer, W. Bras, J. Glossinger, E. Schaible, E. Gann, R. Kirian, A. MacDowell, M. Church, B. Rude, H. Padmore, *Journal of Physics: Conference Series* **2010**, *247*, 012007.

[2] M. Abdelsamie, K. Zhao, M. R. Niazi, K. W. Chou, A. Amassian, *J. Mater. Chem. C* **2014**, *2*, 3373.

[3] R. Noriega, J. Rivnay, K. Vandewal, F. P. Koch, N. Stingelin, P. Smith, M. F. Toney, A. Salleo, *Nat. Materials* **2013**, *12*, 1038.
